# Supplementary figures and images for: Static and dynamic functional connectivity analysis of cerebrovascular reactivity: An fMRI study
Source: Brain Behav. 2020 Apr 27;10(6):e01516. doi: 10.1002/brb3.1516 (PMC7303385; doi:10.1002/brb3.1516)

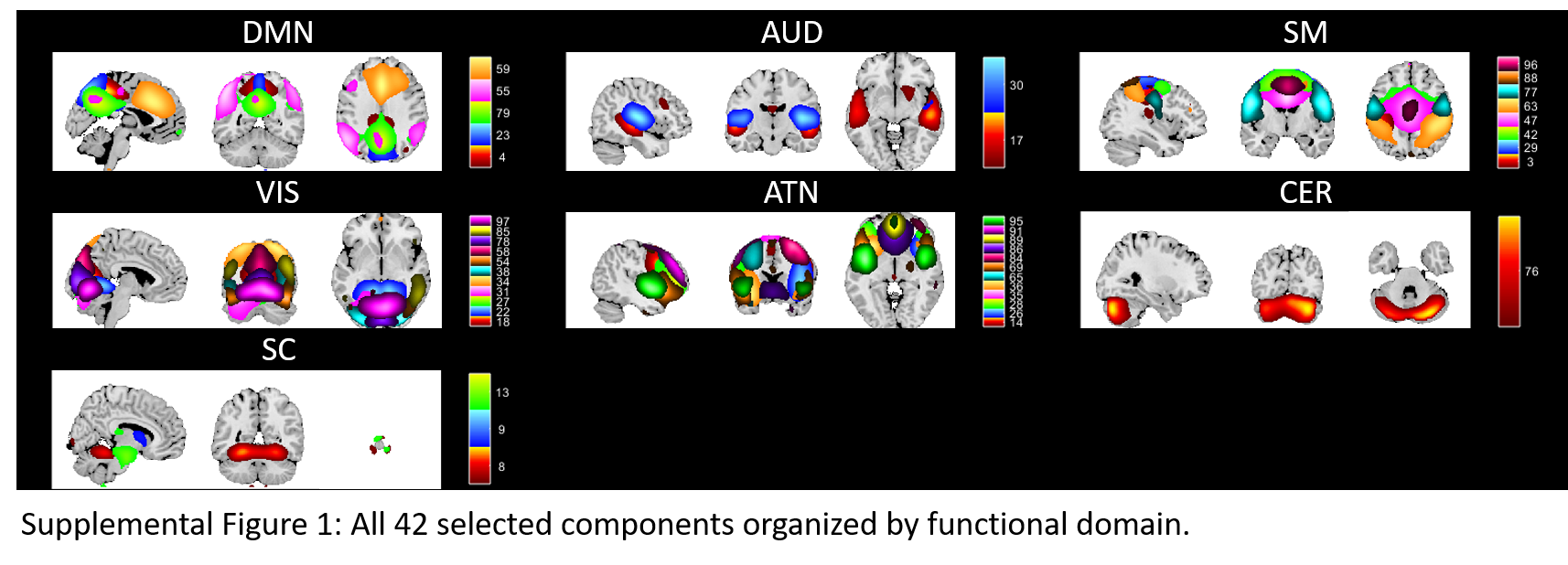

Supplement: Supplementary file 1 [file BRB3-10-e01516-s001.png]
